# An Investigation into Academic Stress and Coping Strategies of South Korean Third Culture Kid (TCK) College Students

**DOI:** 10.3390/bs15030316

**Published:** 2025-03-06

**Authors:** Young-An Ra, Kahyen Shin

**Affiliations:** 1 Department of Psychotherapy, Myongji University, Seoul 03674, Republic of Korea; 2 Counseling and Counselor Education, Syracuse University, Syracuse, NY 13244, USA; kashin@syr.edu

**Keywords:** academic stress, coping mechanism, third culture kids, consensual qualitative research

## Abstract

This study aimed to increase the understanding of academic stress and coping strategies of third culture kids (TCKs) in South Korean colleges. For this aim, six Korean college students who are TCKs were interviewed. For analyzing the interview data, consensual qualitative research was used. As a result, participants’ academic stressors were related to language, interpersonal relationships, learning strategies, career issues, and financial difficulties. As their coping strategies, they reported preparation and review, help-seeking, group study, goal orientation, self-efficacy, and control belief. The results of this study can help South Korean TCK college students with academic stressors, reducing their related stress and allowing them to adjust well in college. We also discussed how educational institutions can help those students overcome academic stress and find their coping strategies.

## 1. Introduction

Third culture kids (TCKs) are individuals who have spent a significant part of their developmental years in a foreign culture due to their parents’ work, mission, or other reasons (S. K. Lee et al., 2022). According to statistics from the Ministry of Education and Human Resources Development, there were 18,024 overseas students and a total of 134,367 students who returned to Korea after studying abroad in the past five years. The population of third culture kids (TCKs) has been steadily growing in recent years, reflecting broader trends in globalization, international migration, and the increasing mobility of families. The interconnectedness of the world has led to an increase in international assignments and job opportunities for individuals and families (Tan et al., 2021). With the expansion of multinational corporations and organizations, there is a rise in international assignments for employees (Caselius & Makela, 2022). These assignments often involve relocating families to different countries, exposing children to multiple cultures and languages (Criel, 2020). Many families relocate to other countries for work, leading to children growing up in cultures different from their parent’s passport country (Donohue, 2024). The proliferation of international schools and educational opportunities catering to expatriate families has contributed to the rise in TCKs (Cheung, 2019). Overall, the growing population of TCKs reflects a broader shift toward global citizenship and cultural diversity. While the TCK experience presents unique challenges, it also offers opportunities for personal growth, cross-cultural understanding, and global perspective-taking.

As TCKs are defined as children who spent part of their developmental years outside their parents’ cultures (Useem & Useem, 1967), they develop a “third culture” that incorporates elements of their parents’ cultures and the cultures in which they have lived (Pollock et al., 2017). They often experience a sense of belonging to multiple cultures while not fully identifying with any single one. TCKs often develop unique skills and characteristics as a result of their upbringing, including adaptability, cultural sensitivity, language proficiency, and a global perspective (Caselius & Suutari, 2023; Wisecarver, 2014). However, they may also face challenges related to identity, belonging, and building long-term relationships due to their mobile lifestyle. Although they have a high level of open-mindedness and cultural empathy, they feel relatively low emotional stability and high loneliness (Dewaele & van Oudenhoven, 2010; Oh et al., 2010). In addition, they have relatively flexible thoughts and actions, can respect the thoughts and actions of others who are different from themselves, and have a high level of tolerance, high international knowledge, and a different perception of being international (Lam & Selmer, 2004). If TCKs develop these characteristics well, there is a good chance that they will grow into the global talent that this era wants. However, TCKs are returning to South Korea for various reasons and are experiencing various difficulties in the readjustment process (A. Jeon, 2021; S. Lee et al., 2018).

### 1.1. Difficulties of TCKs upon Repatriation to South Korea

Unlike general students who live in only one culture, TCKs who have experienced multiple cultures have the characteristic of adapting well to a new culture (Hervey, 2009). TCKs are said to be people who are different from their own and can embrace the cultures of diverse people (Pollock et al., 2017). Due to their experience with two or more cultures, they have strengths such as the ability to adapt to multiple cultures, openness, independence, and high cultural sensitivity (Donohue, 2024; Fanning & Burns, 2017). Research on their intercultural sensitivity also shows that they have a high understanding of other cultures, can respond sensitively to cultural differences, and have a high level of respect for other cultures (Caselius & Makela, 2022; Hayden et al., 2000). Their multicultural experience can serve as an advantage for them, but at the same time, it can also serve as a challenge when returning to South Korea. Y. Lee and Lee (2009) stated that unlike the collectivist and interdependent Korean culture, students who return to South Korea after living in an individualistic culture may experience reverse culture shock in the process of readjusting to Korea’s lifestyle and cultural patterns. They may also show internalization problems such as anxiety, depression, and somatization symptoms (H.-J. Kim et al., 2015). Additionally, in a study conducted by Y. Lee and Lee (2009), the experiences of domestic maladjustment experienced by returning youth included confusion in culture and self-identity. Because they grew up in a relatively free foreign culture, they have difficulty adapting to Korea’s uniform, collective, and authoritarian culture (H. I. Jeon, 2003). In addition, when they return to South Korea, after they have longed for their home country while living abroad, they have a hard time due to the excessive prejudice and indifference of their Korean friends (Kwon, 2018). Because of the negative views toward them, they often are alienated or try to hide the fact that they are students who returned from overseas (Long, 2016; Iem & Shim, 2017). Y. Lee and Lee (2009) studied that returning students felt difficulties with culture and interpersonal relationships in adapting to life in Korea and also felt uncomfortable with changes in their residential environment. Previous studies (Lijadi & van Schalkwyk, 2014; Ra et al., 2024; Rodríguez-Bernal et al., 2023) also mention regarding language difficulties experienced by TCKs. Most TCKs who attended international schools abroad are more familiar with English than Korean, because they lose fluency in one language that they are not using regularly. This can result in a feeling of language disconnect with their home culture and challenges in academic language proficiency (Y. Lee & Lee, 2009; Ra et al., 2024). They also may have unique difficulties such as learning strategies, career prospects, and financial challenges. Thus, TCKs may have specific academic stressors in the South Korean university context. If TCKs passively deal with these difficulties, it can lead to problems such as self-harm or depression (Criel, 2020). Due to this maladaptation, 45% of returnees said they experienced anxiety about returning to Korea, and 9% of returnees expressed complete resistance to returning to Korea (H. M. Kim & Kim, 2014). Therefore, there is a need to look at ways to help TCKs overcome the acculturation difficulties they experience during the readjustment process.

### 1.2. Academic Stress of TCK College Students

Among those difficulties, Berry et al. (1987) stated that education can be a significant factor in lowering acculturation stress in the groups. Through learning, individuals can learn and experience various resources to cope with the experience of acculturation and resolve conflicts. Through high-level learning and achievement, individuals recognize the experience of acculturation as a new challenge and do not accept it as stress (Berry, 2003; Flores et al., 2006; Toth-Bos et al., 2020). Therefore, learning can be said to be an excellent tool that can successfully help individuals adapt to their culture. However, despite this situation, TCKs who have returned home are having difficulty adjusting to school and college.

Academic success during the college years can be a very important developmental task in successfully transitioning to adulthood (DeLaney et al., 2022; Madigan & Curran, 2021). College years can be the initial stage of growing into a mature professional. Thus, academic performance in this period must be developed for a better life (van Linden & Fertman, 1998). In addition, there is also a need to identify the increase in their academic performance for the successful cultural adaptation of many TCK returning college students who are experiencing academic difficulties after returning to their home country. College students find themselves within an environment marked by social transitions and economic changes. This context corresponds with an important life stage characterized by intellectual, career, and psychological development. Academic performance is one of the crucial factors in college students’ campus life. According to Tinto (1975), academic achievement can be a significant predictor of college maintenance and adjustment. College students thus need to find proper and effective learning strategies and ultimately achieve successful academic performance at a university. Previous studies have also emphasized college students’ academic success because it is significantly associated with their interpersonal relationships, as well as career satisfaction after graduation (Bikos et al., 2014; Hawken et al., 2009; Wright et al., 2013). Satisfaction through excellent academic achievement enhances an individual’s self-esteem and plays a major role in self-realization (Kudinov et al., 2020).

However, many TCKs experience a lot of academic stress in Korean colleges (Jarvis et al., 2020; Ra et al., 2024). For TCKs, academic stress is an important factor which stems from subjective psychological distress in many aspects of academic learning and will have a negative impact on their mental health as well as the ability to complete their studies effectively (Akgun & Ciarrochi, 2003). Many South Korean college students have reported serious stress relating to their academic performances because Korean society asks them to be high achievers (Jarvis et al., 2020). High academic achievement in college has been an essential factor for job competitiveness, especially after graduation, because the recent economic crisis in South Korea has increased unemployment (Han & Lee, 2020). Therefore, South Korean TCK college students also experience more academic pressure, stress in their studies, and psychological difficulties (Y. Lee & Lee, 2009; Ra et al., 2024).

### 1.3. Coping Strategies for Academic Stress

Coping has been theorized as cognitive and behavioral responses that work toward negative life events that enable us to control the environment (Lazarus, 1990). According to the transactional stress coping model developed by Lazarus and Folkman (1984), psychological stress is a particular relationship between the person and the environment that is appraised by the person as taxing or exceeding his or her resources and endangering his or her well-being (Lazarus & Folkman, 1984, p. 19). Therefore, coping is an important instrument that occurs between individuals and their environments.

Endler et al. (1998) identified three types of coping strategies based on this transactional stress coping model; they titled them as follows: task-oriented coping, emotion-oriented coping, and avoidance-oriented coping. Task-oriented coping is a strategy in which an individual tries to manage or change the external environment that triggers stress. Emotion-oriented indicates an individual focuses on regulating the internal emotional response to a stressful situation. Lastly, avoidance-oriented coping, sometimes an unhelpful coping strategy, is a third type, where an individual tries to disengage from a stressful situation (Endler et al., 1998). Many researchers support that effective coping strategies are crucial in managing academic stress. Studies have shown that students who employ task-oriented coping strategies, such as time management and problem-solving, tend to have better academic outcomes and lower stress levels (Struthers et al., 2000). Emotion-oriented coping, which focuses on managing emotional responses, can be beneficial in situations where students need to process their feelings about academic pressures (Kausar, 2010). However, reliance on avoidance-oriented coping, such as procrastination or ignoring academic responsibilities, often leads to increased stress and poorer academic performance (Aina & Hermilia Wijayati, 2019). Understanding and implementing effective coping strategies can help TCK college students navigate academic challenges more successfully, reducing stress and improving their mental well-being. Also, the belief that their effective coping can lead to positive results serves as a strong motivator to study hard (Filgona et al., 2020).

Therefore, the purpose of this study is to identify the academic stress of TCK students who report academic difficulties in South Korean colleges. Again, TCK students’ mental well-being is very important for success in college. Therefore, there is a need to help the students who have problems with academic stress. This study was conducted within a group of TCK college students who reported academic stress in their individual counseling. Aiming to clarify TCK academic stressors and coping strategies, the research question for this study is as follows: what are the academic stressors of TCK college students and their coping strategies?

## 2. Methods

### 2.1. Participants

Participants of the present study were six South Korean TCK college students from a 4-year university. Among the participants, three were male and three were female. Their ages ranged from 21 to 24. Three students were sophomores and three were juniors. The length of stay abroad ranged from 3.5 to 11 years. This study employed a snowball sampling method. All participants reported in the individual counseling with their academic advisors that they had been stressed out due to their academic performance. Therefore, about one-hour, semi-structured, in-person interviews were implemented with the research participants. The interviews were conducted in Korean. Before the interviews, participants received informed consent forms, including confidentiality, and agreed to participate in the study. They were told that their participation in this study is voluntary, so they could decide whether they would like to be interviewed or not. Also, they were told that they had the right to stop their participation and leave the study at any time without any penalties or consequences. As they agreed with the study, interviews were conducted. Participants also consented to the interviews being recorded. The sample interview questions were as follows: what factors have the greatest impact on your academic achievement? What is your academic stress? How do you deal with that stress? The interview questions were based on the previous literature (e.g., Aina & Hermilia Wijayati, 2019; Akgun & Ciarrochi, 2003; Jarvis et al., 2020; Struthers et al., 2020) and reviewed by professionals in the educational psychology field. Table 1 indicates information about the participants in this study.

### 2.2. Data Analysis

Researchers first transcribed the interview data collected from participants. The data then were analyzed using Hill et al.’s (2005) consensual qualitative research method. This study consisted of three members of a research team as well as one auditor. All members of the research team are majoring in psychology. They are researchers who have conducted many qualitative studies and are trained in consensual qualitative research. With the consensual qualitative research procedure, researchers carefully read the transcribed interview data and analyzed the contents line by line. It began with the process of extracting meaningful academic stressors and coping strategies from participants and finding core concepts from the data. This study included the four procedures of consensual qualitative research, which are (1) constructing domains to reflect the interview contents, (2) developing core ideas, (3) cross-analysis, which is the process of agreement among researchers, and (4) checking the result as well as its validity (Hill et al., 2005). Analyzing the data was continued until all researchers agreed with the final results. Concepts were described as general if they appeared in all cases, typical if they appeared in more than half of the cases, and variable if they appeared in less than 50% of the cases (Hill et al., 2005). In the present study, data excerpts from the participants were also categorized into three groups based on their frequency of occurrence. General frequency indicated six cases, typical frequency denoted three to five cases, and variant frequency showed one or two cases. After the data analysis, the results were reviewed by participants again and agreement was obtained regarding the results.

## 3. Results

As a result of the analysis of TCK students’ experiences relating to their academic performance, results were primarily divided into academic stressors and their coping strategies. Language, interpersonal relationships, learning strategies, career, and financial difficulties were in the academic stressor domain; preparation and review, help-seeking, group study, goal orientation, self-efficacy, and control beliefs were contained in the coping strategies domain. Table 2 shows the main concepts and contents of the TCK students’ summarized answers.

### 3.1. Domain 1: Academic Stressor

Within the academic stressor domain, there were five factors, namely language, interpersonal relationships, learning strategies, career, and financial difficulties. Participants mentioned that those variables are significant factors that lead TCK students’ stress on academic performance. Each factor is discussed below.

Language: Participants mentioned that language is important as a factor influencing academic stress. Because they mainly used English abroad, they are less familiar with the Korean language than other domestic college students. They have difficulty studying, and this language barrier appears to be stressful for them.

*“Sometimes, I don’t understand things well in class, there are some words I don’t know in text books, thus it’s difficult to study in Korean unless it’s an English class. If I had been better at Korean, I might not have been this stressed out”.*

<Participant 6>

Interpersonal Relationships: TCK students also reported interpersonal relationships as an academic stressor for their academic performance. They mentioned that they are not sure how to get along with professors in college and how to build relationships with them. They also noted that it was difficult for them to make friends in college.

*“I’m not sure how to work with professors in Korea. I feel like rather than being friendly, I should be polite to them”.*

<Participant 4>

Learning Strategies: Participants mentioned that their learning strategies may not be of much help in studying in Korea. They mentioned that they used to get lost a lot while they are studying because the study methods abroad and in Korea are very different.

*“In Korea, there are a lot of things I have to memorize when I study, and there are also many multiple-choice tests. I’ve mostly taken essay tests before. I thus get stressed out because I’m trying to do something I’ve never done before”.*

<Participant 2>

Career: Participants mentioned that career issues can be another academic stressor for their academic performance. They reported that grades in college are closely linked to their career path after graduation in South Korea; thus, they cannot stop thinking about their career and vocational issues while they are studying.

*“Ultimately, studying hard is also about getting a good job. Because of those thoughts, I feel like I have to do well, so it becomes stressful. I always think that I need to get good grades because grades are related to my job after graduation”.*

<Participant 6>

Financial Difficulties: TCK students also mentioned that they considered financial difficulties as an academic stressor. They said that they always think about their financial situation when they are studying. They said that because their grades are significantly related to scholarships, they should study hard, achieve good grades, and earn scholarships for university.

*“There has been pressure to get good grades because of the scholarship. If I get good grades, I will be able to help my parents financially … There was also the worry that I might have to drop out of school without a scholarship”.*

<Participant 5>

### 3.2. Domain 2: Coping Strategies

Within the coping strategies, there were six variables, namely preparation and review, help-seeking, group study, goal orientation, self-efficacy, and control beliefs. Each related factor is discussed below.

Preparation and Review: Participants mentioned that preparation for a class is an important factor influencing academic achievement. Participants reported that they took the time to preview what they will learn in the next class. They mentioned that they usually read class materials or textbooks to be used in class, looked at what they would learn in the next class, and anticipated the contents in advance. They also reported that a clear expectation about the future class helped them to study. The participants also reported that reviewing the learned contents is also an important variable for their academic achievement. The method of review that they used was mainly a rehearsal of replaying the contents of the lesson in their heads. They reported that preparing for the class in advance and reviewing all the contents they learned are the best strategies to achieve better grades and reduce their academic stress.

*“It is much more comfortable to go to class after studying in advance than to go to class without knowing anything. So I try to prepare the class in advance. Also it’s better to review the class contents in advance rather than worrying about the exam later”.*

<Participant 3>

Help-Seeking: Participants mentioned in the interviews that help-seeking behaviors have a significant influence on academic stress. They reported that asking for help from other people, for instance, professors or peers, is important because they can receive a lot of assistance and crucial information for their academic performance.

*“It’s important to ask others for help with my studies. I think there is a lot of information that can be gained by asking about several things such as contents that I learned or study methods”.*

<Participant 1>

Group Study: Participants also mentioned in the interviews that group study is very effective in dealing with academic stress. They reported that studying and preparing for exams together with their friends is very helpful both academically and emotionally. They noted that group study increased their positive emotions and reduced fear toward exams.

*“Studying together for exams is really helpful. I can immediately ask questions about things you don’t know, and it seem to study more if I work together with my peers … I think the atmosphere of studying together increases my motivation to study”.*

<Participant 5>

Goal Orientation: Participants mentioned in the interviews that the orientation to set goals and achieve them has a great influence on their emotions and academic achievement. They reported that goal-orientation is important because setting and achieving these goals increased their academic motivations and decreased academic stress due to the feeling of happiness when the goals were achieved.

*“I think setting goals and achieving them helps a lot for studying. Even when I’m anxious about the test, I feel better when I think that I’ve achieved my goal”.*

<Participant 3>

Self-Efficacy: Participants noted that relaxing academic stress is also related to their self-efficacy. Students mentioned that the belief toward their abilities and confidence that they can complete their work successfully are important in their studying. They also reported that the effort required to gain self-efficacy is important. They noted that the biggest factor in feeling self-efficacy is making an effort when studying.

*“The confidence that I can do it helps me overcome academic stress and get good grades. I am studying while thinking that I can do it even during the exam”.*

<Participant 2>

Control Beliefs: Participants also mentioned control beliefs as a variable affecting how they cope with their academic stress and their academic achievement. They reported that the belief that there will be good results after studying hard can be motivation to study hard. They also noted that if they think about the positive outcomes they will achieve after hard work, they can study harder with patience.

*“I study with a positive thought that if I go through this difficult time, there will be good results. Imagining myself feeling relieved after everything is over”.*

<Participant 1>

## 4. Discussion

This study was conducted to help TCK college students who are struggling with academic stress by identifying the factors that reduce academic stress and their coping strategies. To achieve our research purpose, we explored the two domains from the interview data collected, namely academic stressors and coping strategies. In this section, we provide detailed insights into each domain and highlight the core ideas that emerged from the research data. To discuss the domain of academic stressors, language, interpersonal relationships, learning strategies, career, and financial difficulty were found as core ideas.

The first core idea of academic stressors was language. The lack of speaking the language can impact their understanding of the class contents and communication in the classroom, risking their successful academic achievement in college (Watkins et al., 2012). Attending local or international schools prior to their return to South Korea, many college TCKs have studied in different languages but not in their passport country language before college (Espada-Campos, 2018; H. M. Kim et al., 2018). While TCKs are asked to speak and study in their passport language at the college level, they reported significant academic stress with understanding the written language in textbooks (Lijadi & van Schalkwyk, 2016; Schmidt, 2017). Especially for South Korean TCKs, the academic Korean language with Chinese characters and idioms made their study more challenging (S. Lee et al., 2018; Ra et al., 2019). Aligning with the current research findings, the previous literature indicated language as an academic barrier that makes it difficult for TCKs to understand the class (Long, 2016; Meinberg Paganini, 2020). Some participants in this study mentioned in their interviews that they found the classes difficult unless they took English classes. According to M. Kim (2015), TCKs reported that they had to limit the number of Korean classes due to the language barrier. In addition to difficulties understanding the language, TCKs even received the bias that their education ability is insufficient due to the class language fluency (Brown et al., 2017).

The second core idea of academic stressors was interpersonal relationships. Strong interpersonal relationships in higher education have been reported as an essential predictor of successful academic achievement (Bikos et al., 2014; Kortegast & Yount, 2016). In particular, relationships with professors and peers in South Korea can be different from what TCKs used to have in other countries where they resided (Kwon, 2018; Choi et al., 2013). For instance, TCKs reported that they rarely have non-academic interactions with professors in South Korea (Ra et al., 2019). Our participants reported that they were not sure how to develop relationships with professors, as they did not want to be seen as rude but friendly. TCKs also represented their anxiety of being bulled (Bikos et al., 2014; S. Lee et al., 2018). In particular, TCKs in South Korea experience unfamiliarity with the hierarchical relationships among professors and older peers (J. Lee et al., 2014; H. I. Jeon, 2003). They were sometimes misunderstood to be boasting about their multicultural experience in the community (Huff, 2021; Quick, 2010). In addition, TCK students tend to make friends faster than domestic students due to their familiarity with meeting new people from diverse socio-cultural backgrounds, whereas non-TCKs often have more extended periods to get to know their peers and develop connections at a slower pace (Quick, 2010). Due to the cultural differences in interpersonal stress, confusion and false impressions from professors and peers can create academic stress among college TCKs.

The third core idea of academic stressors was learning strategies. The educational experiences of TCKs can be diverse, including homeschooling, boarding schools, local schools, international programs, and other educational environments with different curricula and standards (Spangler, 2021). Along with the diverse educational backgrounds of TCK students, they often reported differences in their learning strategies (Bailey & Cooker, 2019). TCKs in this study reported that they had to adapt to new learning strategies in order to achieve good grades. Other research also mentioned how the academic system is so different from the passport country that they had to develop new learning strategies (Long, 2016; Schmidt, 2017). In particular, Korean TCKs indicated that they had to memorize specific words and certain concepts rather than understand the contents holistically (J. Lee et al., 2014). Aligning with what our participants reported, TCKs who used to have discussion classes or essay exams had to adjust their exam preparation to focus more on details to obtain better grades. Some of them reported that they did not like the learning styles in South Korea but had to adjust their learning strategies to receive good grades.

The fourth core idea of academic stressors was related to career. Career issues have been reported as a struggle of TCKs after returning to their passport country (Caselius & Suutari, 2023; H. M. Kim et al., 2018). In Korean society, career issues are understood, as they are closely associated with high GPAs (J.-K. Lee & Kim, 2004), which leads TCK students to feel pressured to have a good GPA in order to have a successful career path (Y. Lee & Lee, 2009). Even though they were uncertain about their career path, TCKs still reported they had increased academic pressure to have good GPAs, as that is something that they can control during college. Our participants also noted that their pressure for academic success is associated with career concerns.

The last core idea of academic stressors was financial difficulty. Financial stability is known to be related to academic performance in college settings (Sanborn et al., 2024; Son & Choi, 2020). Although the financial status of TCKs has not been extensively studied, their economic situations can differ depending on the level of support available from their parents, whose professions vary widely (e.g., military personnel, diplomats, business professionals, and missionaries). The previous literature noted mixed findings on whether TCKs experience financial struggles with school tuition or living expenses depending on fluctuating socioeconomic contexts in the countries TCKs experience (Brewer, 2019; H. Kim, 2021). Our study participants reported that they are pressured to secure internal or external scholarships while they study. Some TCKs had to work part-time jobs to be financially self-sufficient during semesters, limiting their time to fully contribute to their studies. Understanding their parents’ financial situation overseas, TCKs in the current study reported prioritizing their financial status before studying.

The other category of the data, coping strategies, has other core ideas such as preparation/review, help-seeking, group study, goal orientation, self-efficacy, and control beliefs. According to the transactional stress coping model developed by Lazarus and Folkman (1984), preparation/review, help-seeking, and group study can be seen as task-oriented coping, while self-efficacy and control beliefs are seen as emotion-oriented coping. However, some of the literature reported that Korean TCKs avoided taking Korean classes due to the academic pressure of needing to compete with domestic students who speak Korean fluently (M. Kim, 2015); avoidance-oriented coping has not been reported among our participants in this study.

First, one of the core ideas of coping strategies is preparation/review. TCK students noted that the preparation and review of the class contents are effective learning strategies for their academic achievement (Ra et al., 2019). Our participants noted that repeating what they learned in the previous class in their heads and anticipating what they will learn in the next class can be a great help to their academic success. To decrease academic stress, TCKs reported that they usually read textbooks and class materials to be used in class in advance and looked at and anticipated what they would learn in the next class. In particular, J. Lee et al. (2014) reported that TCK students look up the vocabulary in the textbooks before they go to class. Although participants did not mention it explicitly, this preparation behavior may mitigate their language barrier. Preparation for the class helps them reduce their anxious feelings because they can overcome unexpected situations in the class. This coping strategy was reported among our participants, noting that they usually use a clear rehearsal of the previous class, and reviewing the class helps them decrease their anxious feelings. These results are also consistent with the research that preparation and reviewing are the most effective ways to achieve academic success and reduce academic stress (Burns & Sinfield, 2012).

The other core idea, help-seeking, was reported as helpful in having a positive influence on their academic achievement and decreasing associated stress. The previous literature has discussed proactive general help-seeking behaviors among TCKs (Criel, 2020). Similarly, in terms of academic help-seeking, TCK students in our study sought assistance from professors and peers to obtain crucial information for their academic performance, including study methods and detailed class content. Some even requested to take exams in English or other languages (Ra et al., 2019). Although they were not sure how to interact with their professors and peers, TCKs reached out to them to obtain academic insights. By reaching out for help from professors and peers, TCKs were able to get used to the new academic context more efficiently.

Not limited to help-seeking, TCKs also noted group study as the third coping strategy. TCKs of our study reported that group study motivated them better during exam periods because they could receive not only academic but emotional support as well. TCKs were able to develop self-efficacy and mitigate their exam anxiety through group study. TCKs frequently engage in group study sessions as their preferred learning method. This behavior contrasts with non-TCKs in South Korea, who tend to study alone. TCKs often experience anxiety and stress when studying by themselves and find studying with others more efficient. The participants in this study reported that group study helped them control their fear of exams more efficiently.

Goal orientation was the fourth core idea. This goal has been known to be an effective buffer for stress for individuals (De Jong et al., 2020), especially in education settings (Brunzell et al., 2016; Tian et al., 2017). Along with this literature, participants in this study reported that goal orientation to set goals and achieve them has a great influence on academic achievement (Bikos et al., 2014). This means that the sense of achievement that can be felt when a goal is achieved is an important motivation for academic achievement for TCK college students. Students are aware of the ability to voluntarily persist in goal-oriented behavior in spite of various circumstances; that is, even if the goal is difficult to achieve and leads to their fear, a successful result can be obtained if one endeavors diligently and steadily. Motivated by various reasons to set goals, from financial reasons to personal interests (Ra et al., 2019), participants from our study reported positive emotions and self-efficacy when meeting their goals.

Other core ideas identified as coping strategies among TCKs were self-efficacy and control beliefs. Self-efficacy is defined as students’ judgments of their abilities to organize and execute the actions required to achieve academic success, while control beliefs refer to students’ beliefs that their efforts will result in positive outcomes (Bandura, 1986). TCK college students in our study indicated that dealing with academic stress was related to the pursuit of self-efficacy. They reported that believing in their ability to complete their academic journey successfully enhances positive emotions and reduces academic stress. Competence in their academic pursuits was found to boost their self-efficacy, positively affecting their learning (Honicke & Broadbent, 2016).

Additionally, TCK students emphasized the importance of control beliefs as their coping strategies. The belief that their hard work will lead to good grades serves as a strong motivator to study hard (Filgona et al., 2020). Consistent with the literature, our participants noted that anticipating positive outcomes from their efforts encourages them to study diligently, remain patient, and overcome academic stress. Based on their report, they are likely to study more effectively and manage academic stress better when they believe their effort will yield significant learning outcomes.

This study is meaningful in that it raises the need to understand the academic stress of TCK college students and ultimately improve their academic performance. The existing literature has been researched to help those students by focusing on academically excellent students’ learning strategies. However, this study focused on academic stressors and their coping strategies related to academic performance. The results of the present study can be used to provide tailored academic support services, such as language assistance programs, counseling, and mentorship opportunities. Additionally, fostering an inclusive campus environment that values diversity and cross-cultural experiences can positively influence TCK students’ academic environment by enhancing their sense of belonging. Implementing peer support groups and academic workshops focused on effective learning strategies and coping mechanisms can also contribute to better academic outcomes. Moreover, providing exams in different languages to accommodate the diverse backgrounds of TCK students can lower their academic stress. This study can benefit college counselors, professors, peers, TAs, and other professionals in university settings by giving them a better understanding of TCKs’ academic and emotional needs, enabling them to offer more effective support and resources.

This study has the following limitations: First, in the data collection, interviews with participants were conducted in the form of self-reporting. In addition, the participants of the present study were six South Korean TCK college students. Thus, the accuracy and reliability of data can be limited to some extent because they were obtained from the only six participants’ recalled memories. Second, this study could not provide the results with a comparison using quantitative research measures and other non-TCK college students. Also, the present study was conducted targeting students at a university in South Korea, so the external validity of this study may be low. Lastly, conducting interviews in Korean may be a limitation, because the participants were not fluent in Korean. To overcome these limitations, future studies should look into actual learning strategies, not students’ perception or recognition level with larger samples. In addition, it is necessary to conduct a repeated study targeting students from other universities with quantitative research methods to generalize the results.

## 5. Conclusions

This study aimed to explore the academic stress and coping strategies of South Korean TCK college students, emphasizing the unique challenges they encounter due to their diverse cultural backgrounds. The findings reveal that TCKs experience significant academic stress related to language barriers, interpersonal relationships, learning strategies, career concerns, and financial difficulties. However, TCKs are able to navigate these challenges with various coping strategies, including preparation and review, help-seeking, group study, goal orientation, self-efficacy, and control beliefs. By understanding these academic stressors and coping mechanisms, educational institutions can better support TCK students through tailored programs and inclusive environments. Ultimately, addressing the needs of TCK students can enhance their academic success and well-being, contributing to their growth as global citizens.

## Figures and Tables

**Table 1:** Participants’ information.

	Age	Gender	Student Years	Second Culture Country	Length of Stay (y)
1	21	Female	Sophomore	Thailand	8
2	22	Female	Sophomore	USA	10
3	24	Female	Junior	India	6
4	23	Male	Sophomore	China	11
5	24	Male	Junior	USA	8
6	24	Male	Junior	Vietnam	3.5

**Table 2:** Main domains, core ideas, and contents of the results.

Domain	Core Idea	Content	Frequency
Academic Stressor	Language	Lack of proficiency in Korean	General
	Interpersonal Relationships	Interaction with professors	Typical
		Peer relationship	
	Learning Strategies	Memorization	Typical
	Career	Anxiety relating to career	Typical
	Financial Difficulty	Pressure for scholarships	Variant
Coping Strategies	Preparation and Review	Looking at the contents in advance and performing mental rehearsal	Typical
	Help-Seeking	Asking for help from professors and peers	Typical
	Group Study	Peer teaching	Typical
	Goal Orientation	Setting a goal and achieving it	Typical
	Self-Efficacy	Belief in capacity to achieve goal	Typical
	Control Belief	Positive expectation of efforts	Variant

## Data Availability

The data that were used in the present study can be provided by the corresponding author upon reasonable request.
